# AIE-YOLO: Auxiliary Information Enhanced YOLO for Small Object Detection

**DOI:** 10.3390/s22218221

**Published:** 2022-10-27

**Authors:** Bingnan Yan, Jiaxin Li, Zhaozhao Yang, Xinpeng Zhang, Xiaolong Hao

**Affiliations:** School of Electronic Engineering, Xi’an Shiyou University, Xi’an 710065, China

**Keywords:** small object detection, context enhancement, large receptive field, wavelet transform, multi-scale feature fusion

## Abstract

Small object detection is one of the key challenges in the current computer vision field due to the low amount of information carried and the information loss caused by feature extraction. You Only Look Once v5 (YOLOv5) adopts the Path Aggregation Network to alleviate the problem of information loss, but it cannot restore the information that has been lost. To this end, an auxiliary information-enhanced YOLO is proposed to improve the sensitivity and detection performance of YOLOv5 to small objects. Firstly, a context enhancement module containing a receptive field size of 21×21 is proposed, which captures the global and local information of the image by fusing multi-scale receptive fields, and introduces an attention branch to enhance the expressive ability of key features and suppress background noise. To further enhance the feature expression ability of small objects, we introduce the high- and low-frequency information decomposed by wavelet transform into PANet to participate in multi-scale feature fusion, so as to solve the problem that the features of small objects gradually disappear after multiple downsampling and pooling operations. Experiments on the challenging dataset Tsinghua–Tencent 100 K show that the mean average precision of the proposed model is 9.5% higher than that of the original YOLOv5 while maintaining the real-time speed, which is better than the mainstream object detection models.

## 1. Introduction

In recent years, the significant improvement of computer hardware technology and the continuous deepening of deep learning theory in the field of image- and object-detection algorithms based on deep learning have made major breakthroughs, which are widely used in automatic driving, intelligent medical treatment, industrial inspection, and remote sensing image analysis, etc. [1,2,3,4]. However, compared with regular-sized objects, small objects carry less information and are susceptible to background interference, which limits the further development of object detection in the real world.

At present, object-detection algorithms based on deep learning can be divided into two categories. One is the two-stage object detection algorithm represented by R-CNN series [5,6,7]. The other is the one-stage object detection algorithm represented by the You Only Look Once (YOLO) series [8,9,10,11] and SSD series [12,13]. The one-stage detection algorithm has the advantages of fast inference speed and high real-time performance, but the accuracy is slightly lower than that of the two-stage object detection algorithm. As one of the YOLO series models, YOLOv5 with an easy network structure has received extensive attention and application due to its consideration of detection accuracy and detection rate at the same time. However, it is not sensitive to small objects and is easy to miss detect. The main reason for the poor detection performance of YOLOv5 for small objects is that the visual features of small objects are not obvious, and the feature information and position information are gradually lost or even ignored after feature extraction of convolutional neural networks (CNNs), which is difficult to be detected by the network. Secondly, small objects, especially those with a size of less than 32 × 32 pixels, are easy to mix with the background. After feature extraction, a pixel on the feature map covers itself and the surrounding background noise, which further increases the difficulty of detection.

Aiming at the problem of feature loss in the feature extraction process of small objects, multi-scale networks such as Feature Pyramid Network (FPN) [14] used by YOLOv3 and Path Aggregation Network (PANet) [15] used by YOLOv4 or YOLOv5 horizontally fuse low-level features and high-level features, which alleviates the information loss to a certain extent. However, the information on the small objects that has been lost after multiple downsampling and pooling operations cannot be recovered. In recent years, some super-resolution (SR) methods have been introduced to the network to compensate for the information loss of small objects by increasing the feature resolution. The Multi-Task Generative Adversarial Network (MTGAN) [16] recovers detailed information by upsampling small blurred images to large-scale images, which greatly improves the detection performance of small targets. Noh et al. [17] proposed a new feature-level super-resolution method, which employs dilated convolution to make the generated high-resolution target features and the low-resolution features generated by the feature extractor maintain the same receptive field. However, the computational cost of feature extraction in the above network would be expensive. In addition, the context information of the target can be obtained to assist in inferring the location or category of the object by using dilated convolution and pyramid pooling operations in CNNs, such as the network proposed by [18,19], which can greatly increase their receptive fields. However, CNNs are still limited by the local calculation method of the convolution kernel, and cannot obtain the dependencies and global information between remote objects in the image.

In view of the above problems, considering the real-time requirements of the detection network, this paper proposes an auxiliary information-enhanced YOLO (AIE-YOLO) based on YOLOv5. By constructing a context enhancement module (CEM), the network adds auxiliary information that is conducive to identifying small objects, and introduces a wavelet transform method to make up for the small object information lost in the feature extraction process, thereby effectively improving the small object detection accuracy of the network.

To summarize, the contributions of this paper are as follows:

(1) Using the information around the small target to enhance the small object information from the side: We propose a context enhancement module, which can obtain rich context information of small objects by fusing features with different scale receptive fields, thus greatly improving the sensitivity and adaptability of the network to small objects in the image. Considering that the context information extracted by the 21 × 21 large receptive field branch will have a lot of background noise, the skip connection branch is introduced to recalibrate the features in both the spatial and channel directions to obtain the attention feature map, thereby enhancing the feature response of the small object and suppressing the background noise.

(2) Increasing the information of the small target itself to enhance the small object information from the front: Aiming at the problem of feature loss caused by the multiple downsampling and pooling operations of the CNNs for small objects, we take the high- and low-frequency information of the original image separated by wavelet transform to participate in multi-scale feature fusion by using the characteristics of multi-resolution and multi-scale analysis of the wavelet transform, which can enhance the feature expression ability of small objects, and improve the effect of multi-scale feature fusion.

(3) Benefiting from the above improvements, experiments on the challenging dataset TT100K show that the proposed AIE-YOLO network reliably improves the performance of small object detection. In addition, the AIE-YOLO network is more capable of small object detection tasks under complex conditions.

The rest of this paper is structured as follows. Section 2 introduces related works about small object detection, wavelet transform, and the attention mechanism. Section 3 describes the proposed methods in detail. The experimental results and analysis are presented in Section 4. Finally, the conclusions are drawn in Section 5.

## 2. Related Work

### 2.1. Small Object Detection

Small object detection has always been a subject of research difficulty in the field of object detection, and researchers have carried out a lot of research on this topic. Gong et al. [20] believed that the top-down connections between adjacent layers of FPN brought a double impact on the detection of tiny objects. Therefore, a fusion factor is proposed to control the information transferred from deep layers to shallow layers to adapt FPN to the detection of tiny objects, and a significant performance gain is achieved on the TinyPerson tiny object detection dataset. Cui et al. [21] proposed a context-aware block network (CAB Net) to improve small object detection by constructing high-resolution and strong semantic feature maps. CAB exploits pyramid dilated convolution to merge multi-level context information to capture the semantic information of small objects while maintaining high resolution, and achieves an average precision of 78% on the Tsinghua–Tencent 100K (TT100K for short) dataset. Yang et al. [22] used a novel query mechanism to accelerate the inference speed of feature-pyramid-based object detection. Coarse locations of small objects are first predicted on low-resolution features, and then accurate detection results are computed using high-resolution features sparsely guided by the coarse locations. In this way, the information can be obtained from the high-resolution feature maps while avoiding useless calculations for the background area. Guo et al. [23] introduced Transformer into the backbone and neck parts of the network to obtain the global information of features and used the bidirectional feature pyramid network (BiFPN) structure to fuse features of different scales to solve the problems of large-scale changes of steel surface defects and poor detection ability of small defects. The network effectively improves the detection accuracy of steel surface defects.

### 2.2. Wavelet Transform

Wavelet transform can obtain different sub-bands with different frequency characteristics through multi-level decomposition, which has the characteristics of multi-resolution and multi-scale analysis. Because it can restore the original signal through inverse transform without loss, it is often used in the fields of signal processing and image analysis.

In recent years, researchers have carried out a lot of research on the combination of wavelets and deep learning. Guo et al. [24] proposed a deep wavelet network for super-resolution tasks, which combines information complementarity in the wavelet domain with deep CNN, and recovers the missing detail information in the process of extracting features using convolution by processing sub-bands. Viewing CNN as a limited form of multi-resolution analysis, Fujieda et al. [25] utilized wavelet transform to compensate for the missing spectral information in the feature extraction process that is beneficial to image processing tasks and integrated it into the CNN architecture, achieving higher accuracy with fewer trainable parameters on texture classification and image annotation tasks. In order to solve the problem that the receptive field size and computational efficiency of CNN cannot be balanced, Liu et al. [26] proposed a novel multi-level wavelet CNN (MWCNN) model, which embeds the wavelet transform into the CNN architecture to reduce the resolution of feature maps. MWCNN can effectively restore the detailed texture and clear structure of the image.

### 2.3. Attention Mechanism

Since small objects are easily disturbed by background noise, researchers propose an attention mechanism to guide the model to focus on the object area and suppress background noise to enhance the feature extraction ability of the network. The essence of the attention mechanism is to assign weight coefficients to the values in the feature map tensor, and assign higher weights to key areas.

Jie et al. [27] proposed a channel attention mechanism, which dynamically strengthens the features of each channel by constructing an SE module to assign weights to the channel dimensions of the feature map, and effectively improves the representation quality of the network. Woo et al. [28] proposed an attention mechanism CBAM that combines the channel attention mechanism and the spatial attention mechanism in a tandem manner, and recalibrates the features in both channel and spatial directions to obtain more comprehensive and reliable attention information. Currently, self-attention mechanism has swept the field of computer vision, such as ViT [29], DETR [30], etc., owing to the ability to establish global dependencies on their own inputs. However, the self-attention mechanism treats 2D images as 1D sequences, which destroys the crucial 2D structure of the image. The quadratic complexity is too expensive for high-resolution images such as remote sensing and traffic signs, which can only capture spatial adaptability but ignores the adaptability in channel dimension.

## 3. Materials and Methods

YOLOv5 has been widely used in many industrial scenarios due to its advantages of fast detection speed, high detection accuracy, and easy deployment. As a general object detection network, YOLOv5 has not made too many improvements for small objects, resulting in its still poor detection performance on small objects. Therefore, targeted improvements to the network are needed to adapt to small object scenarios. Considering the requirement of real-time detection, YOLOv5 is selected as the baseline network for subsequent improvement in this paper.

### 3.1. AIE-YOLO

**Figure 1:**
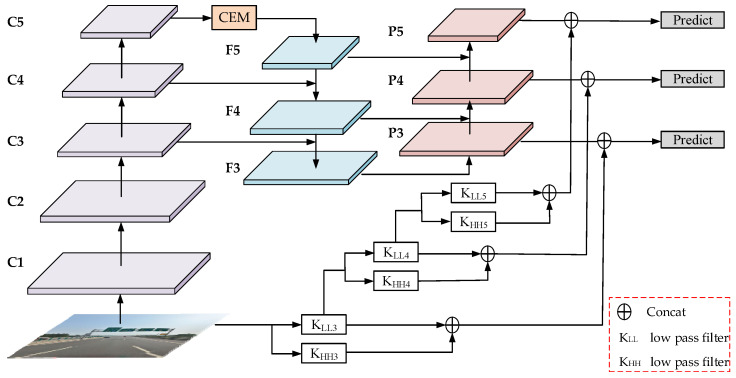
The overall structure of AIE-YOLO network.

The overall structure of AIE-YOLO proposed in this paper is shown in Figure 1. AIE-YOLO consists of four parts: backbone network, CEM, PANet with wavelet transform, and detection head. As we can see in Figure 1, the backbone network is used to extract features from the object image and output feature maps of different scales from shallow to deep. {C1, C2, C3, C4, C5} represent the feature maps after the input image is down-sampled by {2, 4, 8, 16, 32} times, respectively. The CEM is constructed after the highest-level feature map extracted by the backbone network to obtain rich context information on small objects. This module uses the multiway structure to increase the width of the network horizontally to obtain the global and local information of the image, which enhances the coverage and sensitivity of the network for small object feature extraction. In addition, the attention branch is used to improve the ability of background noise suppression.

The high- and low-frequency information obtained from the input image decomposed by the 3th, 4th, and 5th level wavelet transform are involved in the feature fusion of the PANet, thus increasing the visual features of the small object to solve the problem that the features of small objects gradually disappear after the feature extraction of the original YOLOv5. The small object detection is conducted based on the three-way feature map output after feature fusion is completed by the wavelet transform, and the final small object detection result is obtained after the non-maximum value suppression processing.

### 3.2. Context Enhancement Module

In the real world, there is usually a coexistence relationship between “object and scene” and “object and object”, and using this relationship will help improve the detection performance of small objects. For example, after introducing the context information “sky” to the image of a bird whose appearance features are not obvious, the model can identify it as a bird through the coexistence relationship between the sky and the bird.

**Figure 2:**
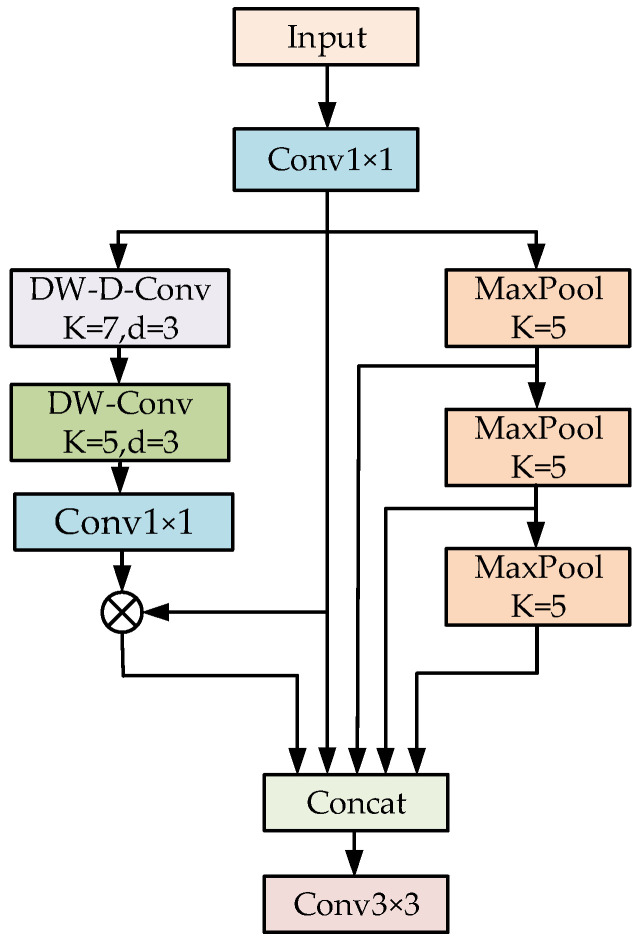
The structure of context enhancement module (CEM).

Therefore, in order to solve the problem of missed detection and false detection of small objects due to the unobvious appearance features, this paper proposes a context enhancement module, which can obtain rich context information of small objects by fusing the features of receptive fields with different scales, and improve the sensitivity and adaptability of the network to small objects in images. The structure of CEM is shown in Figure 2. Firstly, 1 × 1 convolution is used to reduce the dimension of the input channel, and the number of channels is reduced to one half of the original to reduce the number of parameters and calculation of the network. Then feature layers with receptive field sizes of 5 × 5, 9 × 9, 13 × 13, and 21 × 21 are concatenated in parallel with the input map to obtain multi-scale receptive field outputs.

To further improve the efficiency of the structure, three 5 × 5 MaxPools in series are used to obtain feature outputs with receptive field sizes of 5 × 5, 9 × 9, and 13 × 13, where the serial two 5 × 5 MaxPools are equivalent to one 9 × 9 MaxPool, and the serial three 5 × 5 MaxPools are equivalent to one 13 × 13 MaxPool. Considering that the 21 × 21 standard convolution will significantly increase the number of parameters and computation of the network, inspired by MobileNet [31], we propose to decompose a 21 × 21 convolution into a 7 × 7 depth-wise dilation convolution (DW-D-Conv) with a dilation rate of 3, a 5 × 5 depth-wise convolution (DW-Conv), and 1 × 1 standard convolutions. For small objects in complex backgrounds, there will be a lot of background noise in the context information extracted by using large kernel convolutions. Therefore, this paper introduces a skip connection branch to perform an element-wise multiplication operation to enhance the feature response of small objects and suppress background interference, which captures the long-range relationships with slight computational cost and parameters.

The calculation process of the CEM can be expressed as:

(1) $$W_{1}=(f_{conv}^{1\times 1}(f_{DW-D-conv}^{7\times 7}(f_{DW-conv}^{7\times 7}(f_{conv}^{1\times 1}(F))))⊗f_{conv}^{1\times 1}(F))$$

(2) $$W_{2}=f_{\max pool}^{5\times 5}(f_{conv}^{1\times 1}(F))$$

(3) $$W_{3}=f_{\max pool}^{5\times 5}\left(f_{\max pool}^{5\times 5}(f_{conv}^{1\times 1}(F))\right)$$

(4) $$W_{4}=f_{\max pool}^{5\times 5}\left(f_{\max pool}^{5\times 5}\left(f_{\max pool}^{5\times 5}\left(f_{conv}^{1\times 1}(F)\right)\right)\right)$$

(5) $$Y=f_{conv}^{1\times 1}(Cat(W_{1},W_{2},W_{3},W_{4},f_{conv}^{1\times 1}(F)))$$

Here, $f_{conv}^{1\times 1}$ represents a regular convolution operation with a kernel size of 1 × 1, $f_{DW-Conv}^{5\times 5}$ represents a 5 × 5 depth-wise convolution operation, and $f_{DW-D-Conv}^{7\times 7}$ represents a 7 × 7 depth-wise convolution with a dilation rate of 3. ⊗ means element-wise product; Cat represents the feature map concatenation operation; F is the input feature map; $\;W_{1}$ represents a feature map whose output receptive field size is 21 × 21; $W_{2}$, $W_{3}$, and $W_{4}$ represent feature maps with output receptive field sizes of 5 × 5, 9 × 9, and 13 × 13, respectively; and Y denotes the new feature map after context enhancement.

### 3.3. PANet with Wavelet Transform

In deep neural networks, shallow feature maps have the disadvantages of a small receptive field, weak semantic information, and lack of contextual information. However, they have more spatial and detailed feature information, which is beneficial to object localization. The deep feature maps have a larger receptive field and stronger semantic information, which is more suitable for object classification tasks. YOLOv5 adopts the PANet structure as a multi-scale feature fusion module to bidirectionally fuse shallow features and deep features, thus increasing the semantic information of shallow feature maps. To a certain extent, it alleviates the problem of information loss, but it cannot recover the information that has been lost after the downsampling and pooling operations.

Therefore, for the problem above, we use the characteristics of multi-resolution and multi-scale analysis of wavelet transform to integrate the high- and low-frequency information separated by wavelet transform into a multi-scale features fusion to enhance the features of small objects. Specifically, the high-frequency information decomposed by the wavelet transform is beneficial to enhance the detailed features such as texture edges, and the low-frequency information can supplement the global information of the feature map.

In this paper, the discrete wavelet transform of the 2D Haar wavelet transform is used to decompose the original image x into four sub-band images to obtain the high- and low-frequency information involved in the subsequent multi-scale fusion. The specific implementations of wavelet transform participating in multi-scale feature fusion are as follows:

After the input image has undergone 3th, 4th, and 5th level wavelet transform, the feature maps with resolutions of 80 × 80, 40 × 40, and 20 × 20 are obtained, which are consistent with the resolutions of {P3, P4, P5} (As shown in Figure 1). Then, we concatenate the high- and low-frequency information of each level decomposed by wavelet transform to {P3, P4, P5}. Each level of decomposition operation is: four filters ($f_{LL}$, $f_{LH}$, $f_{HL}$, $f_{LL}$) with fixed parameters and a stride of 2 are used to decompose the original image x to obtain four sub-band images of $x_{LL}$, $x_{LH}$, $x_{HL}$, and $x_{HH}$, where the four sub-band images represent the decomposed low-frequency image, vertical detail image, horizontal detail image, and diagonal detail image, respectively. The Haar wavelet filter is:

(6) $$f_{LL}=\left[\begin{array}{cc} 1 & 1\\ 1 & 1 \end{array}\right],\;f_{LH}=\left[\begin{array}{cc} -1 & -1\\ 1 & 1 \end{array}\right],\;f_{HL}=\left[\begin{array}{cc} -1 & 1\\ -1 & 1 \end{array}\right],\;f_{HH}=\left[\begin{array}{cc} 1 & -1\\ -1 & 1 \end{array}\right]$$

The input image is x(i,j), where i is the row and j is the column, then the 2D DWT can be expressed as:

(7) $$\left\{ \begin{array}{l} x_{LL}=x(2i-1,2j-1)+x(2i-1,2j)+x(2i,2j-1)+x(2i,2j)\\ x_{LH}=-x(2i-1,2j-1)-x(2i-1,2j)+x(2i,2j-1)+x(2i,2j)\\ x_{HL}=-x(2i-1,2j-1)+x(2i-1,2j)-x(2i,2j-1)+x(2i,2j)\\ x_{HH}=x(2i-1,2j-1)-x(2i-1,2j)-x(2i,2j-1)+x(2i,2j) \end{array}\right.$$

In order to further strengthen the edge information of the object, Equation (8) is used to significantly enhance the three high-frequency sub-bands of $x_{LH}$, $x_{HL},\;$ and $x_{HH}$ at the same level as the low-frequency sub-band, $x_{LL}$, to obtain the enhanced comprehensive high-frequency sub-band $K_{HH}$. The low frequency sub-band of the same level is denoted as $K_{LL}$, and the final high- and low-frequency information involved in feature fusion is $K_{LLi}$ and $K_{HHi}$, where i=3, 4, 5.

(8) $$K_{HH}(i,j)=\sqrt{x_{LH}{\left(i,j\right)}^{2}+x_{HL}{\left(i,j\right)}^{2}+x_{HH}{\left(i,j\right)}^{2}}$$

**Figure 3:**
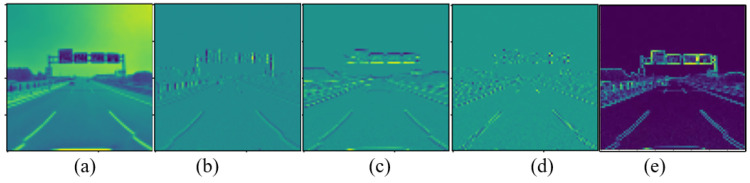
The visual feature map of the traffic sign image before and after reinforcement.

The visual feature maps of the traffic sign image before and after reinforcement are shown in Figure 3. Figure 3a is a low-frequency feature map decomposed by the 3rd level wavelet transform shown in Equation (7). Figure 3b–d are the vertical high-frequency feature map, the horizontal high-frequency feature map, and the diagonal high-frequency feature map after the decomposition of the 3rd level wavelet transform, respectively. Figure 3e is the comprehensive high-frequency feature map obtained by the above-mentioned three high-frequency feature maps through Equation (8). It can be seen that the high-frequency information (texture features) after wavelet transform are further enhanced. The distinguishable features of small objects are added, so that the detection performance of small objects can be improved.

## 4. Experimental Results and Analysis

### 4.1. Experimental Datasets and Evaluation Metrics

**Figure 4:**
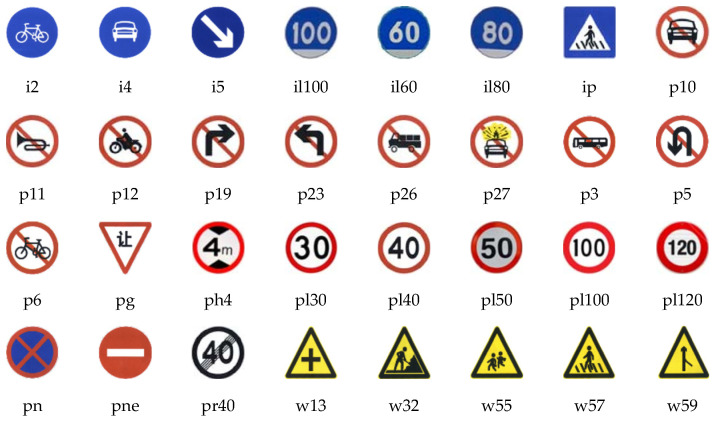
Some traffic sign categories.

In order to verify the effectiveness and reliability of the proposed method, this paper uses the traffic sign dataset TT100K jointly published by Tsinghua University and Tencent for experiments. The dataset contains 30,000 traffic sign instances from 100,000 high-resolution images (with a resolution of 2048 × 2048). A total of 92% of the instances cover less than 0.2% of the whole image, which is easily blocked by trees and is seriously affected by light and weather conditions. It is an excellent dataset for small object detection in complex scenes. Since the number of instances of each category in this dataset is unbalanced, and some traffic signs are not common in real scenes, we refer to [32,33] to select 45 categories with more than 100 instances for experiments. Figure 4 shows 20 common categories among them. The dataset provides a total of 9176 images with full annotation information. This paper selects 7427 of them as the training set, 826 as the validation set, and 917 as the test set, and each part is completely independent. In addition, the annotation information of other categories of the dataset is deleted to improve the robustness of the model.

The mean average precision (mAP), frames per second (FPS), and parameters are used as evaluation indicators. The mean average precision is the average of the average precision (AP) of all categories, which is used to evaluate the detection accuracy of the model. Usually, the metric for judging the speed of object detection is the number of frames per second (FPS), i.e., the number of images that can be processed per second. The larger the value of FPS, the better the real-time performance of the algorithm. The complexity of the model is evaluated by the number of parameters.

### 4.2. Experimental Environment and Parameter Settings

In this paper, all the experiments were performed using a 12th Gen Intel(R) Core(TM) i7-12700 with 16 GB RAM on a platform of a Windows 10, 64 bit, operating system, and NVIDIA GeForce GTX 3090 graphic card having 24 GB of VRAM. The experimental simulation uses the PyTorch deep learning framework. The development environment is Python 3.7, PyTorch 1.11.0, CUDA 11.3.

The size of the network input image is set to 640 × 640 pixels. The optimization algorithm used for model training is Stochastic Gradient Descent (SGD). The batch size is set to 16. Moreover, a momentum of 0.937 is used to adjust network parameters, while a decay weight of 0.0005 is utilized to prevent overfitting. The initial learning rate is 0.01, and the models are trained up to 300 epochs. Cosine annealing strategy is used to adjust the learning rate.

### 4.3. Ablation Study

To verify the effectiveness of the CEM and wavelet transform method proposed by AIE-YOLO on the small object detection task, this paper uses YOLOv5 as the baseline model to carry out a series of ablation experiments on the TT100K testing set. The experimental results are shown in Table 1. As shown in the table, the mAP of AIE-YOLO is 84.8%, which is 9.5% higher than the baseline YOLOv5, and the speed is only slightly decreased (from 119.8 FPS to 100.7 FPS). It shows that the network proposed in this paper can effectively improve the average accuracy of small object detection on the basis of ensuring real-time speed.

Each module improved in this paper has a certain improvement in the detection accuracy of the network. The CEM brings major improvement (from 75.3% to 85.6% mAP), which assists in enhancing the deep semantic information of the small object. The wavelet transform method improves mAP by 6.6% compared with the baseline. It can be observed from the experimental data that, by introducing the high- and low-frequency information decomposed from the original image into PANet to participate in multi-scale fusion, the wavelet transform method could supplement the information lost after the downsampling and pooling operations of the small object, and enhances the network’s ability to locate small objects. The two proposed modules both increase the number of parameters and calculation of the model to a certain extent, so the detection speed decreases, but the decrease is small.

Several image samples including the sparse distribution, dense distribution, and background interference of small objects in the traffic scene are selected to compare the detection results between the original YOLOv5 and the AIE-YOLO proposed in this paper.

**Figure 5:**
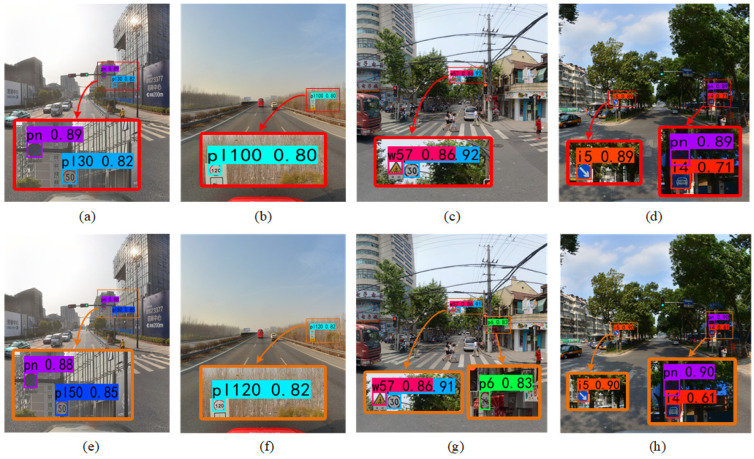
The visualization results: (**a**–**d**) YOLOv5; (**e**–**h**) AIE-YOLO.

The visual detection results are shown in Figure 5. Examples (a)–(d) are the detection results of the baseline YOLOv5, and examples (e)–(h) are the detection results of AIE-YOLO. For better visualization, this article shows a screenshot of the traffic sign area marked by a red rectangle.

Comparing Figure 5a with Figure 5e, it can be found that the proposed method accurately detects the “pl50” and “pn” traffic signs, while the baseline YOLOv5 has false detection in the recognition of “pl50” small objects. This false detection behavior is also reflected in Figure 5b, where the baseline YOLOv5 falsely detected “pl120” as “pl100”. Comparing Figure 5c with Figure 5g, it can be seen that Figure 5c shows that the baseline YOLOv5 can correctly detect larger objects when the distribution of small objects is relatively dense, but miss a small object of “p6”. The AIE-YOLO proposed in this paper correctly detects all the objects in the picture. It can be observed that AIE-YOLO can reduce the probability of false detection and missed detection. The reason is that the context enhancement module obtains the characteristic information of the object and its adjacent areas, as well as the addition of high- and low-frequency information decomposed by the wavelet transform, which plays an important complementary role in the detection of small objects.

Figure 5d,h show the detection results with background interference. It can be observed that both baseline YOLOv5 and AIE-YOLO can correctly detect traffic signs. The comparison found that the detection accuracy of the “i5” category small object and the “pn” category small object are comparable, but AIE-YOLO has higher detection accuracy for the “i4” category small object which is disturbed by the background noise. The results demonstrate that AIE-YOLO can improve the detection effect of small objects disturbed by the background, owing to the use of an attention branch which has the ability to enhance the feature response of the small object and suppress the background noise. In summary, the actual detection results show that AIE-YOLO is more capable of small object detection tasks under complex conditions than YOLOv5.

### 4.4. Performance Comparison of Each Network on the TT100K Testing Set

To further explore the performance of the AIE-YOLO network, this paper reproduces the detection results of five mainstream object-detection networks of Fast R-CNN, RefineDet [34], SSD, YOLOv3-SPP, and YOLOv4 on the TT100K dataset, and compares them with AIE-YOLO and baseline YOLO. The comparison results are shown in Table 2. The bold font in the table indicates the best index results. It can be seen from Table 2 that the mAP of AIE-YOLO is 84.8%, which is 11.7% higher than the two-stage object detection algorithm Faster-RCNN, and 20.7% and 18.1% higher than the single-stage object detection algorithms RetinaNet and SSD, respectively. Compared with the YOLOv3-SPP and YOLOv4 of the YOLO series, the improvement is 28.4% and 23.3%, respectively. Meanwhile, the AIE-YOLO network produces the highest AP in most categories, such as “p6” and “w55” which are most common for small object instances.

The experiments show that AIE-YOLO can obtain the context information around the small object by expanding the receptive field, and in particular, the 21 × 21 receptive field branch can capture the global features that are helpful for small object recognition. At the same time, an additional element-wise multiplication operation can obtain the attention feature map to prevent small objects from being overwhelmed by noise. In addition, the use of wavelet transform can supplement the rich detailed information of small objects, which helps to better locate and classify small objects lacking information in complex scenes, and can effectively improve the detection accuracy of small objects. Faster R-CNN and SSD methods achieve the highest AP in certain categories of detection: Faster R-CNN achieves 89.3% and 91.5% AP for the “ip” and “pl100” categories, respectively, and SSD achieves 88.4% AP for the “il100” category. The mAPs are still slightly lower than AIE-YOLO. In addition, in the object categories with a small number of instances, such as “pl70”, “w32”, and “wo”, AIE-YOLO achieved better AP values of 74.1%, 55.4%, and 50.1%, respectively. This shows that AIE-YOLO can fully mine the feature information of small objects when the number of objects is small.

The inference speed of all the above networks is evaluated on a single Nvidia 3090 GPU with batch size 1. RetinaNet, YOLOv4, and YOLOv5 train the models with the input size 640 × 640. The input size of Fast R-CNN is 1000 × 600, while other approaches have a smaller input size 512 × 512 or 608 × 608. The detection accuracy and speed of each model on the TT100K test set are shown in Table 3. It can be seen from Table 3 that the detection speed of our method is slightly lower than YOLOv5, but far faster than other mainstream object detection networks. Even if the image input resolution is lower than Fast R-CNN, the mAP and speed of AIE-YOLO still have a strong competitive advantage, which verifies the effectiveness of the method in this paper.

## 5. Conclusions

Aiming at the characteristics of small objects with less pixel information and weak feature representation, we propose the AIE-YOLO network to improve the detection accuracy of small objects. The network obtains the global information and local information of the image by constructing the CEM, thus adding deep semantic information which is beneficial to improve the accuracy of small object classification. In addition, an attention feature map is generated to prevent small objects from being overwhelmed by noise in large receptive fields. At the same time, in order to solve the problem that the features of small objects gradually disappear after the downsampling and pooling operations, the wavelet transform is introduced to PANet for improving the effect of multi-scale feature fusion and enhancing the feature expression ability of small objects. On the TT100K data set, AIE-YOLO achieves 84.8% mAP, a 9.5% improvement over the baseline, while reaching a speed of 100.7 FPS. At the same time, it has strong competitiveness compared with other mainstream detection networks.

The results show that the AIE-YOLO network can sacrifice a small amount of speed in exchange for an effective improvement in the accuracy of small object detection, and is more suitable for small object detection tasks in complex environments. Our work provides a reference for research on obtaining higher accuracy for traffic sign detection in autonomous driving. In addition, the methods proposed in this paper can also be used in areas where a large number of small targets need to be detected from the image, such as intelligent medical treatment, defect detection, and aerial image analysis.

## Figures and Tables

**Table 1:** Ablation experimental results.

Baseline	CEM	Wavelet Transform	mAP (%)	Speed (FPS)	Parameters (M)
√			75.3	119.8	7.14
√	√		83.6 (+8.3)	115.1	7.36
√		√	81.9 (+6.6)	102.6	7.73
√	√	√	84.8 (+9.5)	100.7	7.95

In this table, √ means that the corresponding method is adopted.

**Table 2:** AP (part of traffic sign categories) and mAP comparison of different networks on the TT100K testing set.

Method	Backbone	mAP	p6	w55	ip	pl100	il100	pl70	w32	wo	i2	i4	pg	ph4	ph4.5	ph5	po
Fast R-CNN	ResNet-50	73.1	49.3	73.1	**89.3**	**91.5**	84.5	54.3	39.1	41.7	76.2	91.6	87.8	71.9	79.0	46.5	67.7
RetinaNet	ResNet-50	64.1	29.6	65.8	70.0	84.4	73.1	61.1	31.0	34.1	71.6	90.4	90.7	38.0	64.0	33.8	56.7
SSD	VGG16	66.7	26.4	69.7	63.5	88.6	**88.4**	62.5	28.7	39.0	69.4	82.2	83	40.3	77	67.9	47.8
YOLOv3-SPP	Darknet53	56.4	15	33.1	64.6	84	87.1	39.8	34.4	36.1	67.7	78.4	86.2	22.8	73.1	50.8	45.8
YOLOv4	CSPDarknet53	61.5	18.9	30	59.6	88.5	75	60.9	29	30.6	77	93.3	73	22.2	86.1	25.7	51.1
YOLOv5-s	CSPDarknet53	75.3	16.7	80.4	79.1	84.3	84.4	54	22	39.3	**89.4**	94.3	88.2	55.6	79.8	61.8	67.8
AIE-YOLO	CSPDarknet53	**84.8**	**60.2**	**92.3**	83.7	89.9	83.8	**74.1**	**55.4**	**50.1**	87.9	**95.5**	**96.4**	**76.4**	**97.4**	**80.9**	**73.4**

In this table, the best result of each type is bolded.

**Table 3:** Comparisons of accuracy and speed on the TT100K testing set.

Method	Backbone	Input Resolution	mAP (%)	Speed (FPS)
Fast R-CNN	ResNet-50	1000 × 600	73.1	27
RetinaNet	ResNet-50	640 × 640	64.1	24
SSD	VGG16	512 × 512	66.7	63
YOLOv3-SPP	Darknet53	608 × 608	56.4	50
YOLOv4	CSPDarknet53	640 × 640	61.5	52.1
YOLOv5	CSPDarknet53	640 × 640	75.3	119.8
AIE-YOLO	CSPDarknet53	640 × 640	84.8	100.7

## Data Availability

The data presented in this study are available on request from the corresponding author.
